# Overexpression of Bcl-2 induces STAT-3 activation *via* an increase in mitochondrial superoxide

**DOI:** 10.18632/oncotarget.5763

**Published:** 2015-09-27

**Authors:** Jia Kang, Stephen Jun Fei Chong, Vignette Zi Qi Ooi, Shireen Vali, Ansu Kumar, Shweta Kapoor, Taher Abbasi, Jayshree L. Hirpara, Thomas Loh, Boon Cher Goh, Shazib Pervaiz

**Affiliations:** ^1^ Department of Physiology, Yong Loo Lin School of Medicine, National University of Singapore, Singapore, Singapore; ^2^ Cellworks Group Inc., San Jose, CA, USA; ^3^ Cellworks Research India Private Limited, Bangalore, India; ^4^ Experimental Therapeutics Program, Cancer Science Institute, Singapore, Singapore; ^5^ Department of Otolaryngology, National University Healthcare System, Singapore, Singapore; ^6^ NUS Graduate School for Integrative Sciences and Engineering, National University of Singapore, Singapore, Singapore; ^7^ National University Cancer Institute, NUHS, Singapore, Singapore; ^8^ School of Biomedical Sciences, Curtin University, Perth, Australia

**Keywords:** Bcl-2, Rac1, STAT3, superoxide, mitochondria, Autophagy and Cell Death Section

## Abstract

We recently reported a novel interaction between Bcl-2 and Rac1 and linked that to the ability of Bcl-2 to induce a pro-oxidant state in cancer cells. To gain further insight into the functional relevance of this interaction, we utilized computer simulation based on the protein pathway dynamic network created by Cellworks Group Inc. STAT3 was identified among targets that positively correlated with Rac1 and/or Bcl-2 expression levels. Validating this, the activation level of STAT3, as marked by p-Tyr705, particularly in the mitochondria, was significantly higher in Bcl-2-overexpressing cancer cells. Bcl-2-induced STAT3 activation was a function of GTP-loaded Rac1 and NADPH oxidase (Nox)-dependent increase in intracellular superoxide (O_2_^•−^). Furthermore, ABT199, a BH-3 specific inhibitor of Bcl-2, as well as silencing of Bcl-2 blocked STAT3 phosphorylation. Interestingly, while inhibiting intracellular O_2_^•−^ blocked STAT3 phosphorylation, transient overexpression of wild type STAT3 resulted in a significant increase in mitochondrial O_2_^•−^ production, which was rescued by the functional mutants of STAT3 (Y705F). Notably, a strong correlation between the expression and/or phosphorylation of STAT3 and Bcl-2 was observed in primary tissues derived from patients with different sub-sets of B cell lymphoma. These data demonstrate the presence of a functional crosstalk between Bcl-2, Rac1 and activated STAT3 in promoting a permissive redox milieu for cell survival. Results also highlight the potential utility of a signature involving Bcl-2 overexpression, Rac1 activation and STAT3 phosphorylation for stratifying clinical lymphomas based on disease severity and chemoresistance.

## INTRODUCTION

Bcl-2 is the acronym for the B-cell lymphoma/leukemia-2, and as its name implies the gene was first discovered in B-cell malignancies [1]. *Bcl-2* is normally localized to chromosome 18q21, however, its expression is deregulated due to fusion with the immunoglobulin heavy chain gene promoter and enhancer on chromosome 14q32 [t(14,18) chromosomal breakpoint], thereby resulting in overexpression [2]. This observation is associated with drug resistance due to the inherent pro-survival function of Bcl-2 through its ability to block mitochondria-dependent apoptotic signaling [3]. In addition to its canonical activity, our earlier work has demonstrated that overexpression of Bcl-2 impacts mitochondrial redox metabolism *via* an increase in mitochondrial O_2_^•−^ production [4, 5]. The functional relevance of this, from the standpoint of the apoptosis inhibitory activity of Bcl-2, is underscored by the ability of pharmacological or genetic inhibitors of Nox-mediated O_2_^•−^ production to overcome apoptosis resistance in Bcl-2 overexpressing cells [6, 7]. Our recent work implicated the small GTPase Rac1 in Bcl-2-induced O_2_^•−^ production by demonstrating the existence of a physical interaction between the two proteins, as well as the ability of pharmacological and genetic inhibitors of Rac1 to alleviate Bcl-2 induced O_2_^•−^ production and overcome apoptosis resistance [8].

To gain further insight into the functional relevance of the interaction between Rac1 and Bcl-2, we first made use of computer simulation driven predictive experiments based on the protein pathway dynamic network. This simulation platform served as a visualization tool to predict the impact on pathway dynamics upon manipulating Rac1-Bcl-2 interaction in a cancer context. Interestingly, among the targets with high hit scores for positive correlation with Rac1 and/or Bcl-2 expression levels, STAT3 (Signal Transducer and Activator of Transcription 3, was identified. STAT3 is phosphorylated at Tyr705 upon the activation of cytokine and growth factor receptors, resulting in its homodimerization and nuclear translocation to activate transcription of downstream responsive genes [9-14]. STAT3-activated genes not only promote cell proliferation, angiogenesis and metastasis, but also inhibit apoptosis, differentiation and anti-tumor immune responses [10, 12-16]. In addition, a constitutively active form of STAT3 is sufficient for inducing transformation of normal epithelial and immortalized fibroblasts derived from prostate and breast tissues [17]. Active STAT3 is also required in cellular transformation induced by the viral oncogene, *v-src* [10, 15, 17-20]. As such, STAT3 activation is critically involved in the processes of cancer initiation, progression and maintenance [10, 12-15] and elevated levels of active STAT3 are associated with poor prognosis in a host of hematopoietic and non-hematologic malignancies [10, 12, 13, 15, 18, 21]. Therefore, disruption of STAT3 signaling is associated with growth inhibition and apoptosis in cancer cell lines as well as in murine xenograft models of myeloproliferative neoplasms, acute lymphoblastic leukemia, glioblastoma, head and neck squamous cell carcinoma, breast cancer, lung adenocarcinoma and renal cell carcinoma [10, 13-15, 19, 22-28].

Apart from being a transcription factor for many genes with oncogenic potential, such as Bcl-2 [17], what makes STAT3 an appealing candidate to study-based on the predictive screening results from the Cellworks simulation platform-is its involvement in Rac1-driven networks and effects on mitochondrial redox metabolism [29-32]. To that end, GTP-loaded Rac1 has been shown to directly interact with STAT3 via its effector domain and stimulate STAT3 phosphorylation at Tyr705 and Ser727, whereas dominant negative Rac1 inhibits growth factor-induced activation of STAT3 [30]. Interestingly, an additional factor in Rac1-induced activation of STAT3 is intracellular reactive oxygen species (ROS) production; Rac1 is involved in the assembly and activation of NADPH oxidase (Nox) [33-35]. The upstream effect of ROS on STAT3 activation not only involves the activation of Jak2 (Janus kinase 2) and TYK2 (tyrosine kinase 2) that phosphorylate STAT3 [31], but also oxidation-mediated inhibition of the low molecular weight protein tyrosine phosphatase (LMW-PTP) that dephosphorylates Jak2 [32]. Intriguingly, while STAT3 activation is induced upon an increase in intracellular ROS, activated STAT3 itself functions as a stimulus for mitochondrial O_2_^•−^ production [29]. STAT3 has been shown to localize to the mitochondria, where it regulates cellular respiration, and loss of mitochondrial STAT3 suppresses the activities of Complexes I and II of the ETC (Electron Transport Chain) with a resultant decrease in mitochondrial oxygen consumption [29]. The mitochondrial activity of STAT3 appears to share a remarkable degree of similarity with the effect of Bcl-2 overexpression, such as an increase in mitochondrial oxygen consumption and O_2_^•−^ production [4, 5]. Based on these observations, we set out to investigate the crosstalk between cellular redox changes triggered upon Bcl-2-Rac1 interaction, in particular intracellular O_2_^•−^ production, and the activation of STAT3. Furthermore, the translational relevance of these findings was assessed from the standpoint of generating a signature based on the expression and/or activation status of Rac1, Bcl-2 and STAT3, which could have implications for stratifying cancers by disease severity and chemoresistance.

## RESULTS

### Cellworks simulation model predictions reveal STAT3 as a downstream signal mediator of Bcl-2-Rac1 interaction

**Figure 1:**
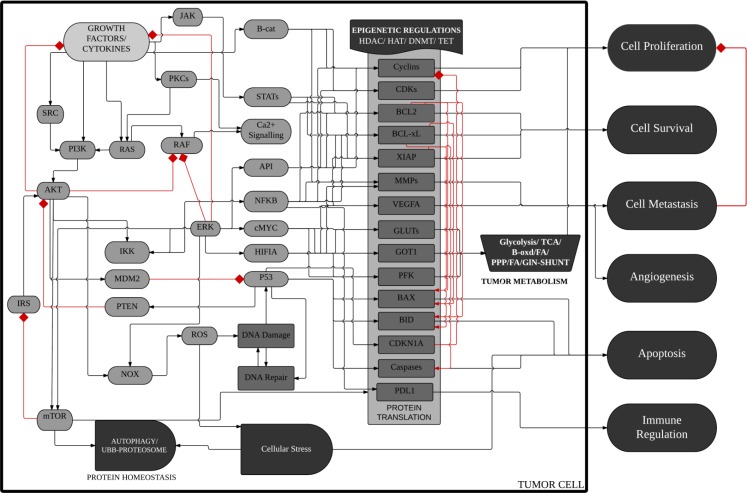
Schematic diagram of ^™^CELLWORKS cancer cell platform A high level schematic diagram of the key pathways, cellular processes and phenotypes in the virtual tumor cell technology. The Cellworks Oncology Platform was customized to create a system aligned to HCT116 human colon cancer cell line (K-Ras mutant, PI3K overexpressed (OE), CDKN2A deleted, β-catenin OE and Bcl-2 OE). Rac1 was overexpressed on the above baseline and Rac1 inhibition was then tested on the above two variants of HCT116. Adapted with permission from Doudican, N. *et al.* [36].

**Figure 2:**
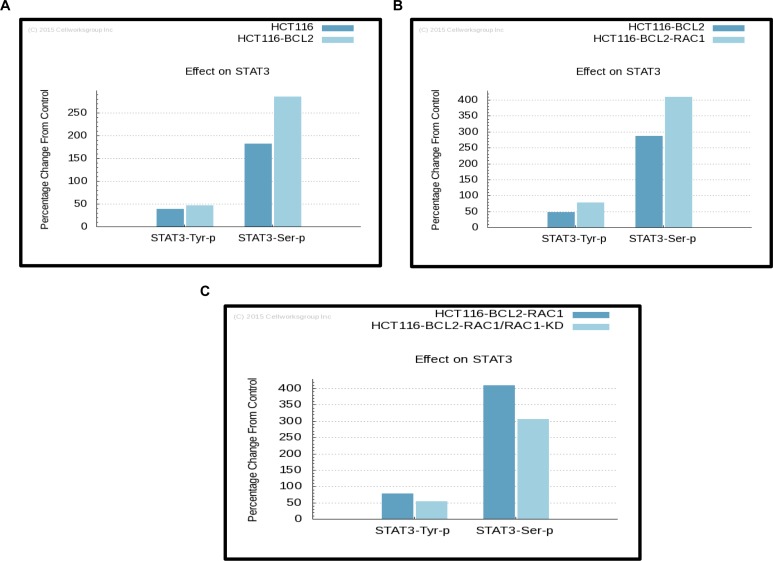
Simulation predictions by ^™^CELLWORKS reveal STAT3 as a downstream signal mediator in cancer cells with Bcl-2 O/E Computer simulation predictive experiments based on the protein pathway dynamic network created by ™CELLWORKS Group Inc. were carried out. The base line used was a K-Ras over-activated, PI3K overexpressed, CDKN2A deleted, β-catenin overexpressed and Bcl-2 overexpressed system aligned to HCT116 human colorectal cancer cell line and a variant of the above base line was created with Rac1 levels overexpressed by 3-fold. Percentage changes on protein levels of phosphorylated STAT3 were predicted in both the base **A.** and variant Rac1 overexpressing cell lines **B.** as compared to the control HCT116 cell line. **C.** Percentage changes in protein levels of phosphorylated STAT3 were predicted upon knocking down of the expression of Rac1 (target inhibition by 70%) in both the base and variant cell lines as compared to the base cell line.

We have previously reported a novel interaction between Bcl-2 and Rac1 and characterized its functional relevance in redox regulation of cell fate and apoptosis resistance [8]. In an attempt to identify the potential downstream mediators of the pro-survival effects of Rac1 and Bcl-2, a large-scale predictive simulation screening was performed on all the proteins that have been documented so far to be involved in tumorigenic signaling (Figure 1; [36]). Interestingly, a significant increase in the phosphorylation of STAT3, in particular STAT3^pSer727^ was predicted upon the overexpression of Bcl-2 in the simulation model (Figure 2A), which was further amplified upon the simultaneous overexpression of Rac1 (Figure 2B and 2C). Furthermore, knocking down Rac1 expression in both the base and variant cell lines resulted in a concomitant decrease in STAT3 phosphorylation levels (Figure 2C). While the effect of manipulating Rac1 on the expression of STAT3 corroborates earlier published work on the interaction between these two proteins [30], the effect of Bcl-2 on STAT3 is intriguing. In terms of the latter, STAT3^pSer727^ has been shown to be involved in the regulation of mitochondrial metabolism [29], and our recent work provides evidence to link Bcl-2 overexpression to an increase in mitochondrial oxygen consumption and O_2_^•−^ production [4, 5]. The effect of Bcl-2 overexpression on other STAT family members was also simulated, which predicted an increase in the total protein levels of STAT5 and STAT6, whereas no effects on STAT1 and STAT4 were observed (Supplementary Figure 1A).

### Bcl-2 overexpression results in a significant increase in STAT3^pTyr705^

**Figure 3:**
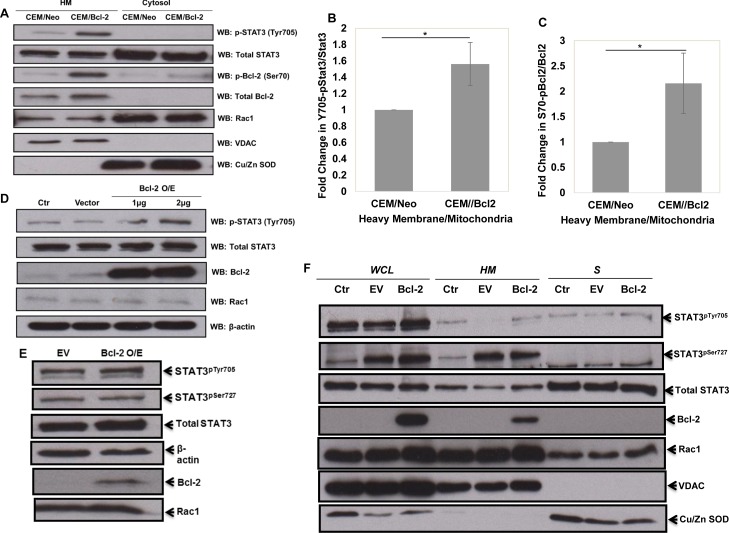
Overexpression of Bcl-2 results in increased STAT3^pTyr705^ **A.** Human chronic myeloid leukemia CEM cells overexpressing Bcl-2 were lysed and fractionated into mitochondria-enriched purified heavy membrane (HM) and cytosolic (S) fractions. The fractions were then probed for STAT3^pTyr705^, total STAT3, Bcl-2^pSer70^, total Bcl-2, Rac1, VDAC and Cu/Zn SOD. **B.** & **C.** Densitometry and statistical analyses of the changing ratios between STAT3^pTyr705^ and total STAT3 as well as Bcl-2^pSer70^ and total Bcl-2 are represented graphically. * denoting *P*-value < 0.05. **D.** & **E.** Lysates from control HeLa cells or cells transiently transfected with either the empty vector or Bcl-2 wild type (1μg of Bcl-2 plasmid was used in E) for 48 hrs were probed for STAT3^pTyr705^, total STAT3, Bcl-2, Rac1, β-actin and STAT3^pSer727^ **E.**. **F.** HeLa cells were either lysed into whole cell lysates (WCL) or fractionated into mitochondria-enriched purified heavy membrane (HM) and cytosolic (S) fractions. The fractions were then probed for STAT3^pTyr705^ & STAT3^pSer727^, total STAT3, total Bcl-2, Rac1, VDAC and Cu/Zn SOD.

Intrigued by the simulation predictive results and the documented involvement of STAT3 in ROS signaling and Rac1 network [29-32], we set out to undertake experiments to validate the simulation predictions *vis a vis* the involvement of STAT3 as a mediator of the pro-survival signaling triggered by Bcl-2-Rac1 interaction. Firstly, the sub-cellular localization of STAT3 was assessed by western blot analysis following subcellular fractionation. Indeed, STAT3 was detected in the cytosolic and mitochondria-enriched HM fractions, and a significantly increased expression of STAT3^pTyr705^ (*P*-value < 0.5) together with Bcl-2 pSer70 (Bcl-2^pSer70^) (*P*-value < 0.5) was detected in the HM fraction of CEM cells overexpressing Bcl-2 (Figure 3A-3C); STAT3^pTyr705^ is an indication of its transcriptional activation [9-14]. We recently reported that the interaction of Bcl-2 and Rac1 was localized at the mitochondria, and that mono-site phosphorylation of Bcl-2 at serine 70 (Bcl-2^pSer70^) stabilized its anti-apoptotic activity and was a poor prognostic indicator in human lymphomas [7]. Interestingly, STAT3 has also been shown to localize to the mitochondria [29]. Corroborating these observations, Rac1 co-localized with Bcl-2^pSer70^ and STAT3^pTyr705^ in the HM (heavy membrane fraction enriched in mitochondria) fraction of CEM/Bcl-2 cells (Figure 3A). To gain further insight into the relationship between STAT3 activation and Bcl-2 expression levels, Bcl-2 was transiently overexpressed in HeLa cells and the activation state of STAT3 (STAT3^pTyr705^) was monitored by western blot analysis. Indeed, transient overexpression of Bcl-2 induced a significant increase in STAT3^pTyr705^ (Figure 3D) but not STAT3^pSer727^ (Figure 3E). Corroborating these results, a significantly higher levels of STAT3^pTyr705^ were detected in the HM fractions of HeLa cells transiently overexpressing Bcl-2, compared to cells transfected with the empty vector (Figure 3F). In addition, while Bcl-2 overexpression did not significantly affect the expression or phosphorylation of STAT1 or STAT5 (Supplementary Figure 1B), a significant induction of STAT1 phosphorylation at Tyr701 was observed upon overexpression of Bcl-xL (Supplementary Figure 1C).

### Bcl-2-induced STAT3 activation is dependent on active Rac1 and intracellular superoxide

**Figure 4:**
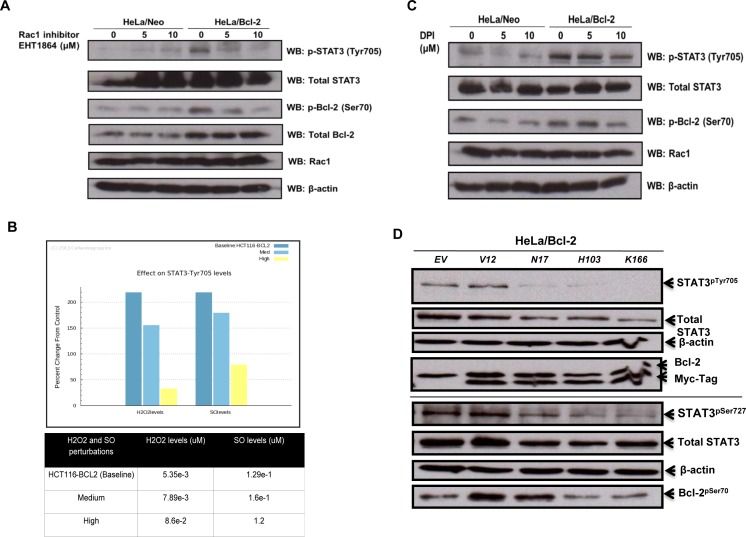
Bcl-2-induced STAT3 activation is mediated by Rac1-dependent O_2_^•−^ **A.** HeLa cells stably overexpressing either the control vector (HeLa/Neo) or Bcl-2 (HeLa/Bcl-2) were treated with different doses of Rac1 inhibitor EHT1864 for 2 hours and the lysates were probed for STAT3^pTyr705^, total STAT3, Bcl-2^pSer70^, total Bcl-2, Rac1 and β-actin. **B.** Percentage change in STAT3^pTyr705^ levels were predicted upon changes in intracellular superoxide levels in the base cell line as compared to the control cell line. **C.** HeLa cells stably overexpressing either the control vector (HeLa/Neo) or Bcl-2 (HeLa/Bcl-2) were treated with the Nox inhibitor DPI for 2 hours and the lysates were probed for STAT3^pTyr705^, total STAT3, Bcl-2^pSer70^, Rac1 and β-actin. **D.** HeLa cells stably overexpressing Bcl-2 (HeLa/Bcl-2) were lysed 48 hours post transient transfection with control empty vector (EV) or various Rac1 mutants (V12, N17, H103 or K166) and probed for STAT3^pTyr705^ & STAT3^pSer727^, total STAT3, Bcl-2^pSer70^, total Bcl-2, Myc-Tag, Rac1 and β-actin in the whole cell lysates.

It has been previously reported that GTP-loaded Rac1 induces STAT3 phosphorylation (Tyr705 and Ser727) and transcriptional activation, whereas dominant negative Rac1 inhibits STAT3 activation induced by growth factors [30]. Coupled to this are our recent findings indicating a role for active Rac1 in the increase in mitochondrial O_2_^•−^ triggered upon overexpression of Bcl-2 [8]. In the light of these reports, we questioned whether the effect of Bcl-2 overexpression on STAT3 activation was a function of intracellular O_2_^•−^ and involved active Rac1. To test that, we first made use of the pharmacological inhibitor of Rac1, EHT1864, which specifically decreases Rac1 activity by locking it in an inactive conformation without affecting protein expression. Pre-treatment of HeLa cells stably overexpressing Bcl-2 with EHT1864 resulted in a decrease in STAT3^pTyr705^, compared to the empty vector matched HeLa/Neo cells (Figure 4A), thus indicating that active Rac1 was required for Bcl-2-induced phosphorylation of STAT3. Interestingly, a dose-dependent decrease in Bcl-2^pSer70^ was also observed with EHT1864 in Hela/Bcl-2 cells (Figure 4A), corroborating our unpublished data (*Kang et al.* unpublished results) implicating active Rac1 in the phosphorylation of Bcl-2 at Ser70.

One of the major functions of active Rac1 is the assembly and activation of Nox family of oxidase to stimulate intracellular O_2_^•−^ production [33-35]. In this regard, not only is Rac1 activation involved in STAT3 phosphorylation, but also intracellular ROS activate STAT3 by either positively regulating the corresponding kinases [31] and/or inhibiting the phosphatase [32]. Before setting out to test this hypothesis experimentally, we ran a simulation using the Cellworks platform where the intracellular levels of O_2_^•−^ and/or H_2_O_2_ were steadily increased in the *in silico* model cell line. The simulation predicted that a mild increase in intracellular O_2_^•−^ or the O_2_^•−^ to H_2_O_2_ ratio significantly induced STAT3^pTyr705^, which understandably decreased upon severe oxidative stress (Figure 4B). Furthermore, treatment with increasing concentrations of DPI, a general pharmacological inhibitor of Nox, resulted in a decrease in the level of STAT3^pTyr705^ in HeLa/Bcl-2 cells (Figure 4C), thereby supporting a probable regulatory role for O_2_^•−^ in Bcl-2-induced STAT3 activation. Of note, the level of Bcl-2^pSer70^ was also significantly decreased upon DPI treatment (Figure 4C), which corroborates our recent report on the redox regulation of Bcl-2 [7]. Next, to gain insight into the functional significance of active Rac1 in Bcl-2-induced STAT3 activation, we employed the various functional mutants of Rac1, namely a constitutively active mutant V12, a dominant negative mutant N17 (threonine to asparagine substitution that has essentially no affinity for GTP), and two functional mutants H103 (alanine substituted by histidine) and K166 (glutamic acid substituted by lysine) that compromise the ability of Rac1 to activate Nox-driven O_2_^•−^ production [37-39]. Notably, transient transfection of HeLa/Bcl-2 cells with the dominant negative mutant N17 resulted in a virtually complete inhibition of STAT3^pTyr705^ as well as STAT3^pSer727^ (Figure 4D).

**Figure 5:**
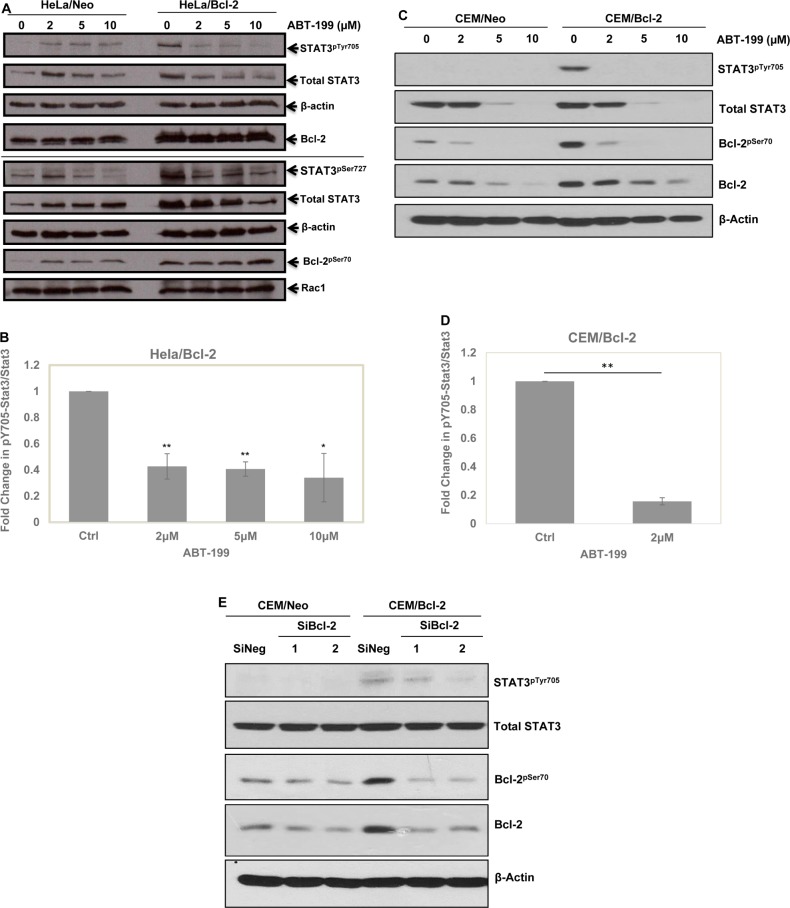
Involvement of Bcl-2, specifically BH3 domain, in STAT3^pTyr705^ **A.** HeLa cells stably overexpressing either the control vector (HeLa/Neo) or Bcl-2 (HeLa/Bcl-2) were treated with different doses of Bcl-2 specific BH3 mimetic ABT-199 for 24 hours and the lysates were probed for STAT3^pTyr705^ & STAT3^pSer727^, total STAT3, Bcl-2^pSer70^, total Bcl-2, Rac1 and β-actin. **B.** Densitometry and statistical analyses of the changing ratio between STAT3^pTyr705^ and total STAT3 in HeLa/Bcl-2 are represented graphically. * and ** denoting *P*-value < 0.05 and < 0.02 respectively. **C.** CEM cells stably overexpressing either the control vector (CEM/Neo) or Bcl-2 (CEM/Bcl-2) were treated with different doses of Bcl-2 specific BH3 mimetic ABT-199 for 24 hours and the lysates were probed for STAT3^pTyr705^, total STAT3, Bcl-2^pSer70^, total Bcl-2, and β-actin. **D.** Densitometry and statistical analyses of the changing ratio between STAT3^pTyr705^ and total STAT3 in CEM/Bcl-2 are represented graphically. ** denoting *P*-value < 0.02 respectively **E.** Effects of Bcl-2 silencing with 2 different SiRNA sequences in CEM/Neo and CEM/Bcl-2 cells on STAT3^pTyr705^ following 48 hours. Lysates were probed for STAT3^pTyr705^, total STAT3, Bcl-2^pSer70^, total Bcl-2, and β-actin.

We have previously implicated the non-structured loop region and the BH3 domain of Bcl-2 in the increase in intracellular O_2_^•−^ induced upon overexpression of Bcl-2 [8]. Utilizing a BH3 mimetic, ABT199, which is highly selective for Bcl-2, unlike other BH3 mimetics that non-specifically target other Bcl-2 anti-apoptotic family members such as Bcl-xL [40], we obtained a significant drop in STAT3^pTyr705^ and STAT3^pSer727^ as well as the total STAT3 levels in HeLa/Bcl-2 and CEM/Bcl-2 cells (Figure 5A-5D). Additionally, we previously showed that knock-down of Bcl-2 in CEM/Bcl-2 cell line could reverse the elevated level of O_2_^•−^ [4]. Using 2 different siRNA sequences to knock-down Bcl-2, we observed a dramatic decrease in STAT3^pTyr705^ (Figure 5E). Together, these results implicate the involvement of Bcl-2 (specifically the BH3 domain) induced increase in intracellular O_2_^•−^ in the downstream activation of STAT3.

### STAT3 activity affects mitochondrial superoxide levels

**Figure 6:**
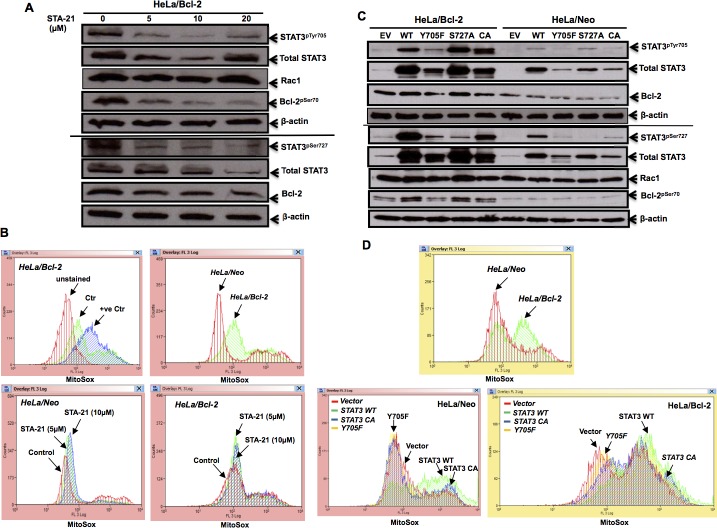
STAT3 activity affects mitochondrial superoxide levels **A.** HeLa cells stably overexpressing Bcl-2 (HeLa/Bcl-2) were treated with different doses of STAT3 inhibitor STA-21 for 24 hours and the lysates were probed for STAT3^pTyr705^ & STAT3^pSer727^, total STAT3, Bcl-2^pSer70^, total Bcl-2, Rac1 and β-actin. **B.** Mitochondrial O_2_^.−^ levels of HeLa/Neo and HeLa/Bcl-2 cells, upon exposure to various doses of STA-21 for 24 hours, were detected by MitoSox staining and analyzed by flow cytometry. At least 10,000 events were analysed by the Summit software. Histograms shown are representative of at least three independent experiments. Authenticity of mitochondrial O_2_^.−^ levels, detected via Mitosox staining, is displayed in supplementary figure S2. **C.** HeLa/Neo and HeLa/Bcl-2 cells were lysed following a 48-hour transient transfection with either the control empty vector (EV), STAT3 wild type (WT) or mutants (Y705F, S727A, CA) and probed for STAT3^pTyr705^ & STAT3^pSer727^, total STAT3, Bcl-2^pSer70^ total Bcl-2, Rac1 and β-actin. **D.** Mitochondrial O_2_^.−^ levels of HeLa/Neo and HeLa/Bcl-2 cells upon transient transfection with either the empty vector (EV), STAT3 wild type (WT) or mutants (Y705F, CA), was detected by MitoSox staining and analyzed by flow cytometry. At least 10,000 events were analyzed by the Summit software. Histograms shown are representative of at least three independent experiments.

It has previously been shown that STAT3 could regulate mitochondrial redox metabolism, independent of its transcriptional activity. Having shown that Bcl-2-induced STAT3 activation was dependent on intracellular O_2_^•−^, we next asked whether the activated STAT3 could, in turn, affect mitochondrial O_2_^•−^ levels, thereby setting up a vicious positive feedback loop. To do so, we first made use of the specific pharmacological inhibitor of STAT3, STA-21, which inhibits the dimerization and DNA binding activity of STAT3 [41]. Indeed, incubation of cells with STA-21 resulted in a significant decrease in STAT3^pTyr705^ and STAT3^pSer727^ as well as total STAT3 in Hela/Bcl-2 (Figure 6A). Interestingly, STA-21 did not have a significantly effect on the increase in mitochondrial O_2_^•−^ in HeLa/Bcl-2 cells (Figure 6B), thereby suggesting that the effect of STAT3 on mitochondrial redox metabolism might be a function independent of its transcriptional activity. To further investigate the link between STAT3 phosphorylation and Bcl-2 induced increase in mitochondrial O_2_^•−^, HeLa/Neo and HeLa/Bcl-2 cells were transiently transfected with: (a) a plasmid carrying STAT3 WT or (b) a plasmid carrying a functional mutant Y705F or (c) a plasmid carrying a functional mutant S727A or (d) a plasmid carrying a constitutively active (CA) STAT3. As expected, the Y705F mutant resulted in a significant decrease in STAT3^pTyr705^; however, a significant decrease in Bcl-2^pSer70^ was also observed upon the transient expression of this functional mutant in Bcl-2 overexpressing cells (Figure 6C). Furthermore, only the transient expression of STAT3 WT or STAT3 CA resulted in an increase in mitochondrial O_2_^•−^ production, while the functional mutant Y705F did not have a significant effect on mitochondrial O_2_^•−^ (Figure 6D). Collectively, these data corroborate earlier findings linking STAT3 to mitochondrial redox metabolism; however, the effect appears to be a function of STAT3^pTyr705^ and not STAT3^pSer727^.

### Bcl-2 expression or phosphorylation correlates with STAT3 activation in clinical lymphomas

Intrigued by our predictive and experimental data linking Bcl-2 overexpression to STAT3 activation, we set out to investigate the clinical relevance of this association in primary cells derived from patients with lymphomas. We performed expression analyses for STAT3^pTyr705^ and STAT3^pSer727^, total STAT3, Bcl-2^pSer70^), total Bcl-2 and Rac1, in lysates obtained from cells isolated from biopsies from 41 patients diagnosed with lymphomas (Supplementary Figure 3A-3I). The respective protein expression levels were normalized to β-actin of that particular blot. Pearson Correlation Coefficient (PCC) analysis was subsequently used to examine potential positive correlation between any two parameters within stratified sub-category of lymphomas. Interestingly, a strong correlation between STAT3^pSer727^ and total Bcl-2 levels was observed in large B-cell lymphomas (*N* = 6) with a correlation coefficient of +0.9339 (Table 1). In the same set of large B-cell lymphomas, the ratio of STAT3^pSer727^ within the total pool of STAT3 also correlated positively (+0.9104) with the ratio of Bcl-2^pSer70^ in the total pool of Bcl-2 (Table 1). Furthermore, a positive correlation between total STAT3 and Bcl-2^pSer70^ was observed in diffuse Large B cell lymphomas (*N* = 4) with a correlation coefficient of +0.9180 (Table 1). Other positive correlations with coefficients less than 0.9 were also obtained in the sub-categories of Hodgkin's lymphomas, B-cell lymphomas, diffuse large B-cell lymphomas and follicular lymphomas, respectively, as summarized in Table 1.

**Table 1:** Correlation between Bcl-2 expression or phosphorylation levels and STAT3 activation status in primary cells derived from patients with lymphoma

	p-Y705 STAT3 vs. p-570 Bcl-2	p-S727 STAT3 vs. Total Bcl-2	Total STAT3 vs. p-570 Bcl-2	Total STAT3 vs. Total Bcl-2	p-Y7OS STAT3/Total STAT3 vs. p-570 Bcl-2/Total Bcl-2	p-S727 STAT3/Total STAT3 vs. p-570 Bcl-2/Total Bcl-2
**Hodgkin's lymphoma (N=7)**	**+0.8318**	-	-	**+0.7851**	**+0.7775**	-
**Large B-Cell Lymphoma (N=6)**		+0.9339	-	-	-	+0.9104
**B-Cell Lymphoma (N=6)**	-	-	**+0.7652**	**+0.7862**	-	-
**Diffuse Large B- Cell Lymphoma (N=4)**	**+0.7646**	-	+0.9180	-	-	-
**Follicular Lymphoma (N=4)**		-	**+0.7132**		-	-

Pearson correlation coefficient statistical analysis was performed between STAT3 and Bcl-2 in sub-categorized lymphoma patient samples.

## DISCUSSION

### Bcl-2-induced STAT3 phosphorylation involves active Rac1 and intracellular O_2_^•−^

In this report we provide evidence to link overexpression of Bcl-2 to the activation of STAT3. Stimulated by our earlier reports linking overexpression of Bcl-2 to an increase in mitochondrial oxygen consumption and O_2_^•−^ production [4, 5], the identification of a novel interaction between Bcl-2 and the small GTPase Rac1 and the inhibitory effect of blocking Rac1 activity on Bcl-2-induced pro-oxidant state [8], and more importantly the ability of intracellular O_2_^•−^ to promote Bcl-2^pSer70^ and stabilize its anti-apoptotic activity [7], here we set out to decipher the downstream mediator(s) of the pro-survival effect of Bcl-2-induced increase in intracellular O_2_^•−^. The predictive simulation pointed to a significant increase in STAT3^pSer727^ upon overexpression of Bcl-2, which was further amplified upon Rac1 overexpression.

Based on the predictive simulation and the observations linking STAT3 activation to cell proliferation, survival, angiogenesis and metastasis [10, 12-16] as well as its involvement in mitochondrial respiration, redox signaling and Rac1 network [29-32], the crosstalk between Bcl-2, Rac1 and STAT3 was explored. Consistent with earlier published report [29], STAT3 was localized to the mitochondria of cells overexpressing Bcl-2. Furthermore, a strong association between STAT3^pTyr705^ and Bcl-2^pSer70^ in the mitochondrial fractions of Bcl-2 overexpressing CEM cells was revealed. This is intriguing given the fact the pTyr705 is a signal for the nuclear localization and transcriptional activation of STAT3, while pSer727 has been shown to translocate to the mitochondria and induce the activity of ETC complexes 1 and II [29]. A recent report also demonstrated that STAT3^pSer727^ localized to the mitochondria in a complex with GRIM19, resulting in mitochondrial ROS generation and necroptosis, upon treatment with TNF-α [42]. However, STAT3^pTyr705^ recruitment to the mitochondria upon overexpression of Bcl-2 that signals for cell survival appears to be a novel regulatory function of STAT3. There are a few interesting observations that provide a mechanistic explanation for this association. Firstly, Bcl-2-Rac1 interaction was identified at the mitochondria [8], which was shown to be responsible for mitochondrial O_2_^•−^ production in cells overexpressing Bcl-2. Secondly, the activity of Rac1 appears important in Bcl-2 mediated increase in mitochondrial O_2_^•−^ [6, 8]. Thirdly, Rac1 has been shown to interact with STAT3 [30] and active Rac1 induces STAT3^pTyr705^ [30], and our unpublished results suggesting that Bcl-2^pSer70^ is critical in its interaction with GTP loaded Rac1 (*Kang et al., unpublished results*). Finally, ROS have been shown to phosphorylate STAT3 by either activating the corresponding kinases [31] and/or inhibiting the phosphatase [32]. Notably, specific inhibition of Rac1 activity, in particular its ability to activate NOX-driven O_2_^•−^ generation, as well as the Bcl-2 BH3 mimetic, significantly rescued the effect of Bcl-2 overexpression on STAT3^pTyr705^ and STAT^pSer727^. The intermediary role of active Rac1 in the phosphorylation of STAT3 upon overexpression of Bcl-2 is also corroborated by the significant inhibition of STAT3^pTyr705^ and STAT^pSer727^ as well as Bcl-2^pSer70^ upon transfection with a dominant negative form of Rac1 (N17) as well as mutants that specifically compromise the ability of Rac1 to activate NOX assembly. Along similar lines, pharmacological inhibition of intracellular NOX with DPI resulted in a dose dependent decrease in STAT3^pTyr705^ as well as Bcl-2^pSer70^ in cancer cells overexpressing Bcl-2. Notably, in a recent report we highlighted the role of intracellular O_2_^•−^ in promoting Bcl-2^pSer70^ and stabilizing its anti-apoptotic activity [7]. Based on these observations, it is plausible that overexpression of Bcl-2 in human cancers promotes the activation of the small GTPase Rac1 and its interaction with Bcl-2, resulting in an increase in intracellular O_2_^•−^ that activates STAT3^pTyr705^ and its mitochondrial recruitment as well as the downstream transcription of target genes involved in proliferation and survival. These findings also suggest the possibility of a potential tripartite interacting complex involved in translating the signal from intra-mitochondrial redox changes to the transcription of target genes involved in tumorigenesis. In this regard, residue 37 in the Switch 1 effector loop region active Rac1 was shown to associate with STAT3 [30], which is also implicated in the interaction of Rac1 with Bcl-2 (*Kang et al. unpublished data*). It is unlikely that Bcl-2 and STAT3 compete for the same binding site on Rac1; however, it is plausible that in cancers with Bcl-2 overexpression, the induced GTP loading onto Rac1 promotes its interaction with STAT3 as well as phosphorylation of STAT3 as a result of redox changes or induced conformational change. Exploring these possible interactions and their functional relevance will be the focus of our future investigations.

### STAT3^pTyr705^ induces mitochondrial *O_2_^•−^* production and Bcl-2^pSer70^

It has been previously reported that STAT3, in particular STAT3^pSer727^ induces the activity of complexes 1 and II of the mitochondria electron transport chain [29]. A recent report also demonstrated that STAT3^pSer727^ localized to the mitochondria in a complex with GRIM19, resulting in mitochondrial ROS generation and necroptosis, upon treatment with TNF-α activation in cells overexpressing Bcl-2 [42]. Interestingly, a significant decrease in Bcl-2^pSer70^ was also observed upon functional inhibition of STAT3. Furthermore, introduction of a plasmid carrying the WT STAT3 mimicked the effect of Bcl-2 overexpression in terms of mitochondrial O_2_^•−^ production in Hela/Neo cells, thereby supporting a role for active STAT3 in mitochondrial redox metabolism. These findings point to the existence of a positive feedback loop, whereby overexpression of Bcl-2 drives mitochondrial O_2_^•−^ production through the intermediacy of active Rac1, which induces phosphorylation/activation of STAT3. The activated STAT3 not only induces the expression of *Bcl-2*, but also amplifies mitochondrial O_2_^•−^ production that triggers Bcl-2^pSer70^, thus keeping Bcl-2 in a conformation amenable for interaction with Rac1 and maintaining its anti-apoptotic activity. Along these lines, inhibition of upstream signaling that triggers STAT3 activation or gene knockdown of *STAT3* sensitized human breast cancer cells to chemotherapy by compromising the anti-apoptotic activity of Bcl-2, thus providing support to our hypothesis that STAT3-Bcl-2 axis serves as a pro-survival mechanism and contributes to the acquisition of drug resistance phenotype [43].

### Positive correlation between STAT3 and Bcl-2 expression in clinical lymphomas

Not only has STAT3 activation been shown to trigger signaling networks involved in proliferation and survival, but a recent report provides convincing evidence that activation of STAT3 signaling is an important surrogate network activated in oncogene addicted cancer cells as a means to evade drug-induced execution [44]. Therefore, inhibition of the STAT3 feedback loop is emerging as a promising strategy to overcome chemoresistance. To that end, STAT3 inhibitors, OPB-31121 and OPB-51602, are being evaluated in phase I clinical trials for advanced solid tumors [45, 46], and a dual inhibitor of STAT3 and NF-κB, RTA 402, is currently under phase II clinical testing in patients with solid tumors and lymphoid malignancies [47]. The identification of STAT3 as a downstream mediator of the pro-survival effect of Bcl-2-Rac1 interaction that involves a critical role of cellular redox status reinforces the significance of STAT3 activation in carcinogenesis and its progression. Analyses of protein expressions in lysates of primary cells derived from patients with clinical lymphomas, albeit limited numbers (*N* = 41), reveal interesting sub-type specific associations, such as the strong correlation between, (a) STAT3^pSer727^ and total Bcl-2 expression in Large B cell Lymphomas, (b) Bcl-2^pSer70^ and total STAT3 levels in Diffuse Large B cell Lymphomas, (c) STAT3^pTyr705^ and Bcl-2^pSer70^ in Hodgkin's Lymphoma. Along similar lines, we recently reported that a higher ratio of Bcl-2^pSer70^ to superoxide dismutase 1 (SOD1) was associated with disease severity or poor outcome in human B cell lymphomas [7]; SOD1 is involved in the regulation of intracellular O_2_^•−^. In light of these observations, it is tempting to speculate that a signature of Bcl-2^pSer70^, STAT3 phosphorylation (STAT3^pTyr705 and/or pSer727^), active Rac1, and low SOD1 (indicative of higher O_2_^•−^) may be associated with disease severity and/or poor outcome in patients with lymphomas and other hematopoietic malignancies or could be beneficial in stratifying malignancies where Bcl-2 overexpression presents a therapeutic challenge. Furthermore, a therapeutic strategy combining BH3 mimetics [40, 48] with STAT3 inhibitors could have potential implications in cancers where one and/or both signaling networks are deregulated.

## MATERIALS AND METHODS

### Primary cells from patient-derived biopsies and cell lines

Primary tumor tissues were obtained from patients at National University Hospital (NUH), Singapore, in accordance with approved IRB protocol. Mononuclear cells and lymphocytes were isolated by Ficoll Hypaque centrifugation as described previously [49]. The human cervical carcinoma HeLa cells were obtained from American Type Culture Collection (ATCC, MD, USA). CEM and HeLa stably transfected with pcDNA3 vector containing either the neomycin gene (HeLa/Neo) or the human Bcl-2 gene (HeLa/Bcl-2) as well as HeLa (ATCC) cells were cultured in RPMI-1640 and DMEM media supplemented with 10% FBS, 1% L-glutamine and 1% PS. The stable clones were maintained in 20 μg/ml of selective antibiotics G418.

### Cellworks simulation cancer model

The Cellworks *validated* simulation technology comprehensively models signal transduction, epigenetic regulation, protein homeostasis pathways and machinery including proteasome and autophagy, metabolic pathways and all other relevant regulations representing all cancer phenotypes. To account for contradictions in published research, the model is built and constantly enhanced through manual scientific interpretation, aggregation and representation. Functional proteomics data based on various publications known to date are combined together with engineering technologies and methodologies (such as interconnecting ordinary differential equations that describe enzyme kinetics, pathway flux distribution, *etc*.). It is a platform that mimics the human physiology system and allows the visibility into how pathways/phenotypes respond/change in a dynamic fashion upon certain stimuli/manipulation. The predictive results are subsequently validated through wet lab experiments, thus greatly reduce the time and cost needed for the initial large-scale screening required otherwise [50]. The Cellworks Oncology Platform was customized to create a system aligned to HCT116 human colorectal cancer cell line with K-Ras mutation, PI3K overexpression (OE), CDKN2A deletion, β-catenin OE and an additional Bcl-2 OE. A variant cell line was also created with Rac1 OE based on the above baseline to study the effect on two phosphorylated forms of STAT3 (Tyr705 and Ser727) along with the impact of RAC1 and/or Bcl-2 knock down on this variant. The effect of increasing levels of intracellular O_2_^•−^ (SO) and H_2_O_2_ was also observed on STAT3 phosphorylation levels.

### cDNA plasmids, siRNA, antibodies and chemical reagents

Bcl-2 and its control vector pcDNA3 as well as Bcl-xL and its control vector pBabe plasmids were a kind gift from Dr. Elizabeth Yang (Vanderbilt University, USA). Bcl-2 mutant S70A and S70E were generated by our laboratory using QuikChange®Site-Directed Mutagenesis Kit (Stratagene, CA, USA). The Rac1 mutants V12, N17, H103, K166 and control vector pIRES plasmids were a kind gift from Dr. Marie-Veronique Clément (Department of Biochemistry, National University of Singapore, Singapore). The wild type STAT3, mutants Y705F, S727A, CA and the control vector pcDNA3 were kindly provided by Dr. Fu Xin Yuan (Cancer Science Institute, National University of Singapore, Singapore). Bcl-2 SiRNA sequences 1 (Hs_BCL2_9) and 2 (Hs_BCL2_10) were obtained from QIAGEN, Venlo, Netherlands. Goat anti-mouse IgG horseradish peroxidase (HRP) and goat anti-rabbit IgG HRP were obtained from Pierce, IL, USA. Goat anti-VDAC, mouse anti-myc-tag, mouse anti-STAT3, rabbit anti-p-Bcl-2 (Ser70), rabbit anti-STAT1 and anti-STAT5, rabbit anti-p-STAT1 (Tyr701) and anti-p-STAT5 (Tyr694) antibodies, rabbit anti-Cu/Zn SOD, rabbit anti-p-STAT3 (Tyr705 & Ser727) antibodies were obtained from Cell Signalling Technology, MA, USA. Mouse anti-β-actin, mouse anti-Bcl-2, mouse anti-Bcl-xL, rabbit anti-Bcl-2 antibodies were obtained from Santa Cruz Biotechnology, CA, USA. Mouse anti-Rac1 antibody was obtained from Upstate, NY, USA. Diphenyleneiodonium chloride (DPI) and superoxide dismutase bovine (Bovine-SOD) were obtained from Sigma-Aldrich, LO, USA. EHT1864 was obtained from Tocris Bioscience, Bristol, United Kingdom. STA-21 was obtained from Santa Cruz Biotechnology, CA. All other chemicals were from Sigma Aldrich, unless otherwise stated.

### Subcellular fractionation by differential centrifugation

Cell pellets (20 × 10^6^ cells) were resuspended in 500μl of extraction buffer A and incubated at 4°C for 20 mins, followed by Dounce homogenization. The homogenate was centrifuged at 300 g for 10 mins at 4°C. The supernatant was additionally centrifuged at 20000 g for 30 mins (fractions enriched with intact mitochondria as marked as HM). The supernatant from the last centrifugation was spun again at 100,000g for 45 mins to obtain the cytosolic fraction. Immunoblotting with VDAC and Cu/Zn SOD antibodies was carried out to show the purity of mitochondria and cytosolic fractions, respectively.

### Measurement of mitochondrial superoxide levels

Cell pellet was resuspended in 100μl plain RPMI-1640 medium with 10μM MitoSox dye (Invitrogen, CA, USA) and incubated in the dark at 37°C for 15 min as described previously. Excess dye was then washed away twice with 1X PBS and the pellet was resuspended in 500μl plain RPMI-1640 medium. At least 10,000 events were analyzed by flow cytometry (Epics Elite EPS, Beckman Coulter, FL, USA) and data were further analyzed using WINMDI software.

### Transient overexpression and knock-down

#### Superfect transfection of HeLa cell line

0.25 × 10^6^ cells/well were seeded one day prior to transfection. On the day of transfection, 2μg plasmid was mixed with 200 μl of plain DMEM medium and 8 μl of SuperFect reagent (Qiagen, Venlo, Netherlands). The mixture was vortexed for 10 secs, incubated at 37°C for 10 mins, topped up with 300 μl of DMEM medium and then added into the wells and incubated at 37°C for 3 hrs. The cells were then washed three times with 1X PBS and incubated in fresh DMEM medium.

#### Electroporation-based silencing of CEM cell line

The Neon^TM^ Electroporation transfection system (Invitrogen, Eugene, OR, USA) was used to transfect SiRNAs into CEM/Neo and CEM/Bcl-2 cell lines. Approximately 10 million cells were harvested, pelleted at 800 rpm and washed with 1X Dubelco's Phosphate-Buffered Saline (PBS containing NaCl, Na2PO4 and KCl but not Ca2+ and Mg2+) prior to re-suspending in Resuspension Buffer R, provided by the manufacturer. 100nM of siRNA sequence was then mixed with the suspended CEM cells and was loaded into a 100μl Neon tip. CEM cells were then transfected with the SiRNA via the Neon electroporation system at 1150 V/30 ms for 2 pulses. Following transfection, CEM cells are transferred to a 100 mm cell culture dish filled with 10 ml antibiotic-free RPMI-1640 supplemented with 2mM L-glutamine and 10% FBS. Cells were then cultured at 37°C/5% CO_2_/95% humidity for 48hours prior to harvesting and western blot analysis.

### SDS-PAGE and western blot analysis

Cells were lysed using RIPA lysis buffer containing protease and phosphatase inhibitors. An average of 60 μg of total protein per sample was subjected to 12% SDS-PAGE. Following wet transfer of the resolved proteins, the polyvinylidene fluoride (PVDF) membrane was incubated with blocking buffer for 1hr on a shaker. After that, the membrane was washed with TBST three times (10 mins each) and probed with desired primary antibody in TBST overnight on a shaker at 4°C. On the following day, the membrane was again washed three times and incubated with the respective secondary antibody in TBST with 1% non-fat milk on a shaker for 1hr. Finally, after three washes the membrane was exposed to SuperSignal West Pico Luminol/Enhancer Solution and Stable Peroxide Solution (Pierce, IL, USA) for the protein bands to be visualized (Medical X-ray Processor, Kodak, NY, USA).

## SUPPLEMENTARY MATERIAL FIGURES


