# Supplementary material for: Overexpression of Bcl-2 induces STAT-3 activation via an increase in mitochondrial superoxide
Source: Oncotarget. 2015 Sep 27;6(33):34191–205. doi: 10.18632/oncotarget.5763 (PMC4741445; doi:10.18632/oncotarget.5763)
Supplement: Supplementary file 1 [file oncotarget-06-34191-s001.pdf]

Overexpression of Bcl-2 induces STAT-3 activation *via* an increase in mitochondrial superoxide

Supplementary Material

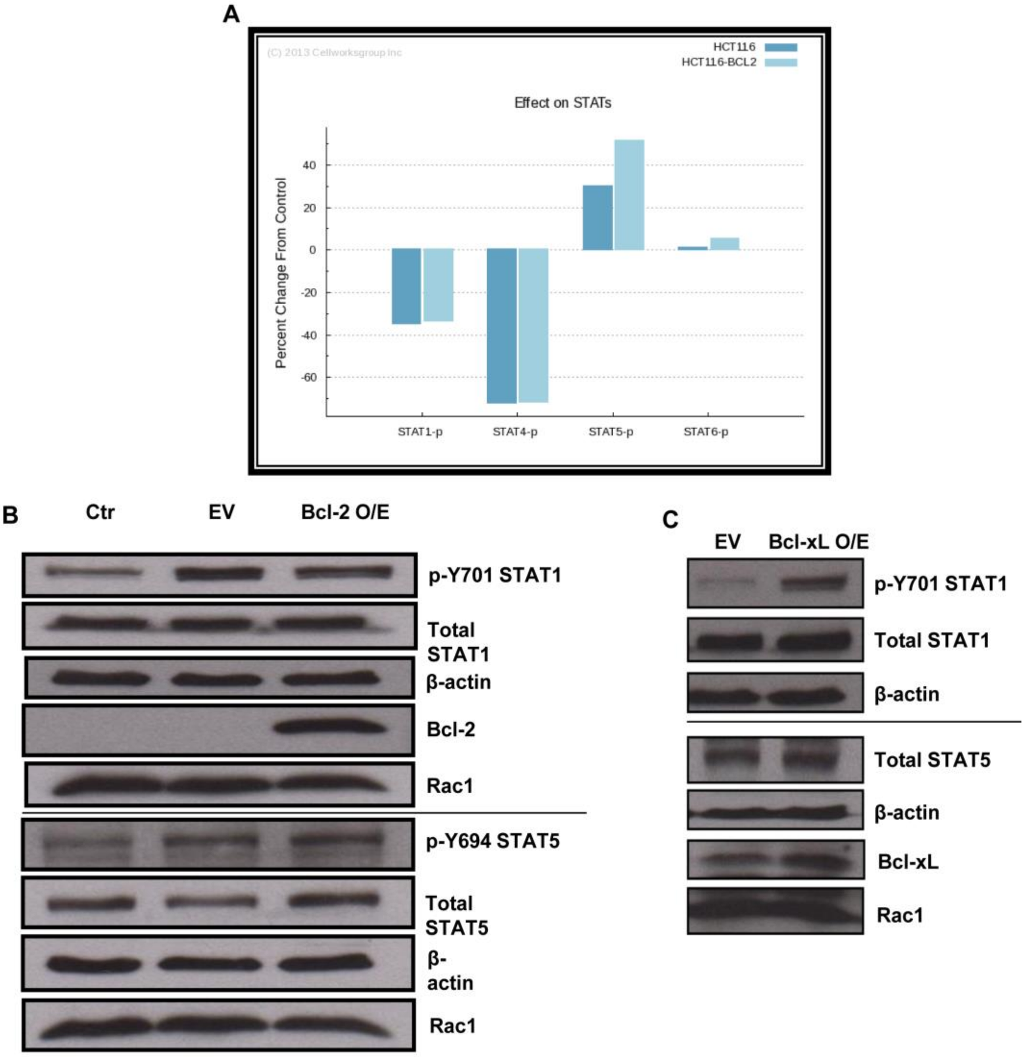

Supplementary Figure 1: Correlation of Bcl-2 and Bcl-xL with other STAT family members. (A) Percentage changes on protein levels of STAT1, 4, 5 and 6 were predicted in both the base and variant cell lines as compared to the control HCT116 cell line. (B) Lysates from control HeLa cells or cells transiently transfected with either the empty vector or Bcl-2 wild type for 48 hrs were probed

for p-STAT1 (Tyr701), total STAT1, p-STAT5 (Tyr694), total STAT5, Bcl-2, Rac1 and β-actin; EV stands for empty vector control. (C) Lysates from HeLa cells transiently transfected with either the empty vector or Bcl-xL wild type for 48 hrs were probed for p-STAT1 (Tyr701), total STAT1, total STAT5, Bcl-xL, Rac1 and β-actin.

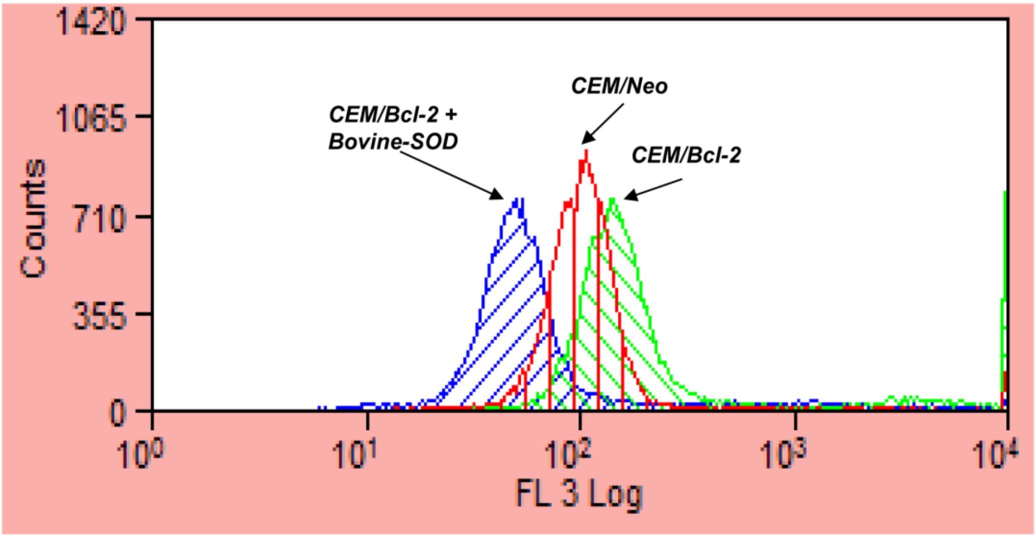

MitoSox

Supplementary Figure 2: Bcl-2 overexpression increases mitochondrial superoxide in CEM cell line. Mitochondrial $O_2^{\cdot -}$ level of CEM/Bcl-2 cells treated with Bovine-SOD (1000unit) was detected by MitoSox staining and analyzed by flow cytometry. At least 10,000 events were analysed by the Summit software. Histograms shown are representative of at least three independent experiments.

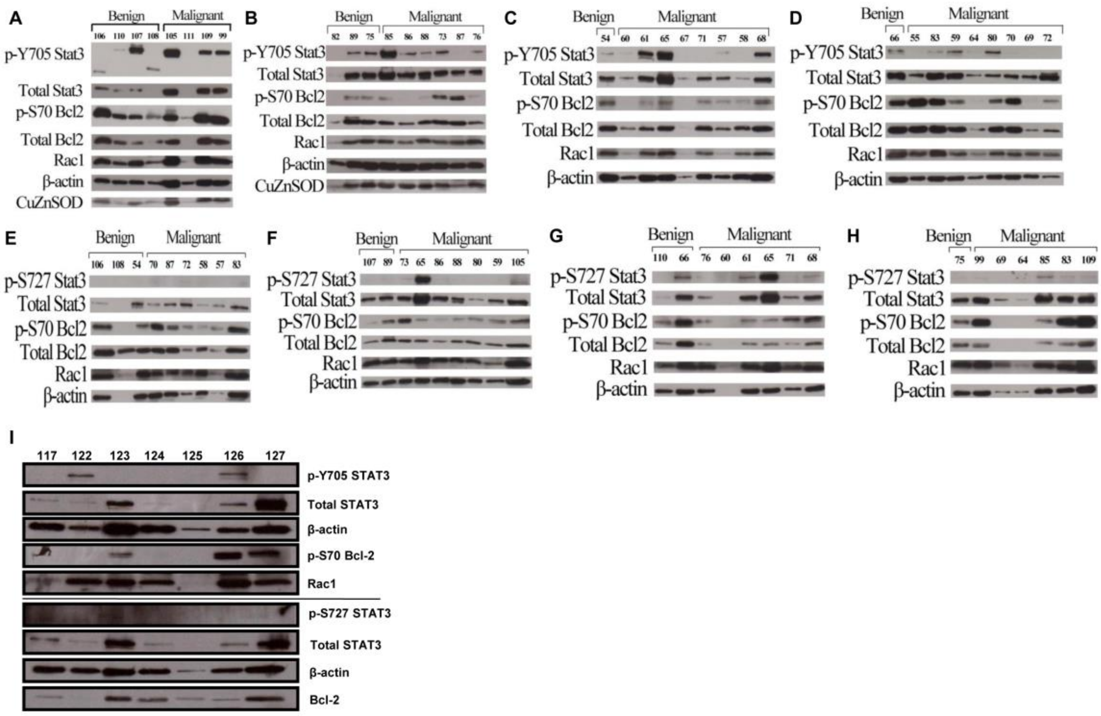

Supplementary Figure 3: Screening of protein expression levels in lymphoma patient samples. A)-I) Western blot analysis of protein expression levels for $STAT3^{pTyr705}$ & $STAT3^{pSer727}$, total STAT3, $Bcl\text{-}2^{pSer70}$, total Bcl-2, Rac1, Cu/ZnSOD and β-actin in lymphoma patient samples. The diagnosis for each patient is summarized as follows: 54,75,123 Reactive lymphoid hyperplasia; 55,83,109,126 Follicular Lymphoma; 57,58,68,125 Diffuse large B-cell lymphoma; 59,64,80,85,86,88,111,122 Hogkin's lymphoma; 60,61,65,67,71,76 Large B-cell lymphoma; 66 Benign lymphadenopathy; 69 T-cell lymphoma; 70,73 Mantle cell lymphoma; 72,87,99,117, 124,127 B-cell lymphoma; 105 Non-Hogkin's lymphoma; 107 Reactive T-lympoid; 110 Accumulation of lipid material; 106 Fibrous Sear; 89, 108 Necrotizing Granulometous Inflammation; 82: Reactive lymphadenitis.
